# Local atomic structure modulations activate metal oxide as electrocatalyst for hydrogen evolution in acidic water

**DOI:** 10.1038/ncomms9064

**Published:** 2015-08-19

**Authors:** Yu Hang Li, Peng Fei Liu, Lin Feng Pan, Hai Feng Wang, Zhen Zhong Yang, Li Rong Zheng, P. Hu, Hui Jun Zhao, Lin Gu, Hua Gui Yang

**Affiliations:** 1 Key Laboratory for Ultrafine Materials of Ministry of Education, School of Materials Science and Engineering, East China University of Science and Technology, Shanghai 200237, China; 2 Key Laboratory for Advanced Materials, Centre for Computational Chemistry and Research Institute of Industrial Catalysis, East China University of Science and Technology, Shanghai 200237, China; 3 Institute of Physics, Beijing National Laboratory for Condensed Matter Physics, Chinese Academy of Sciences, Beijing 100190, China; 4 Beijing Synchrotron Radiation Facility, Institute of High Energy Physics, Chinese Academy of Sciences, Beijing 100049, China; 5 School of Chemistry and Chemical Engineering, The Queen's University of Belfast, Belfast BT9 5AG, UK; 6 Centre for Clean Environment and Energy, Gold Coast Campus, Griffith University, Queensland 4222, Australia

## Abstract

Modifications of local structure at atomic level could precisely and effectively tune the capacity of materials, enabling enhancement in the catalytic activity. Here we modulate the local atomic structure of a classical but inert transition metal oxide, tungsten trioxide, to be an efficient electrocatalyst for hydrogen evolution in acidic water, which has shown promise as an alternative to platinum. Structural analyses and theoretical calculations together indicate that the origin of the enhanced activity could be attributed to the tailored electronic structure by means of the local atomic structure modulations. We anticipate that suitable structure modulations might be applied on other transition metal oxides to meet the optimal thermodynamic and kinetic requirements, which may pave the way to unlock the potential of other promising candidates as cost-effective electrocatalysts for hydrogen evolution in industry.

## Abstract (web summary)

Catalytic activity can be modulated by tuning local atomic structure. Here, the authors show that tungsten trioxide, which is usually inert, can be converted into an efficient electrocatalyst for hydrogen evolution in acidic media, and claim that this could be due to the tailored electronic structure.

## Graphical abstract

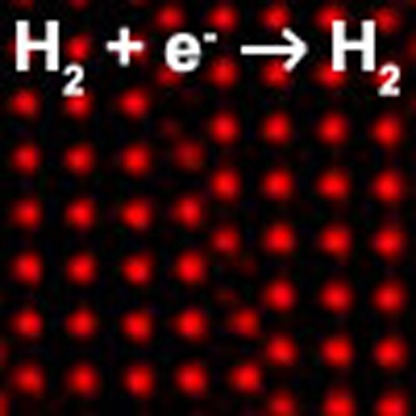

Hydrogen, when generated directly from water, would be a promising chemical fuel for sustainable energy applications^1^,^2^,^3^,^4^,^5^. Development of hydrogen evolution reaction (HER), 2H^+^+2e^−^→H_2_, has attracted worldwide interest^1^,^6^,^7^,^8^. Platinum (Pt) can effectively catalyse the electrochemical reduction of protons in acidic media to form molecular hydrogen at low overpotentials^9^, which remains as the most electrocatalytically active catalyst, but its high cost and low abundance limit large-scale commercial application of electrocatalytic hydrogen evolution^10^. Thus, efficient Pt-free catalysts are highly desired for facilitating the global scalability of such potential clean energy technology. However, as a large and important class of chemical compounds, most transition metal oxides fail to electrocatalyse hydrogen evolution in acidic water, although numerous carbides, nitrides, phosphides and sulfides have shown the capacity for this reaction^11^,^12^,^13^,^14^.

As an important oxide, tungsten trioxide (WO_3_) is much more thermodynamically stable in acidic electrolyte than most metal oxides, and it has attracted intense research interests owing to its potential applications in a wide range of fields such as catalysis, photoelectrochemical cells, photochromic devices and gas sensors^15^,^16^,^17^,^18^. Moreover, recent reports have manifested that WO_3_ can act as the support of noble metals and in itself possessing electrocatalytic activity for hydrogen evolution^19^,^20^,^21^. Unfortunately, the adsorption energy of the atomic hydrogen on W-site is undesirable, leading to the poor activity of WO_3_ for HER in acidic media. Noteworthy, as the descriptor of catalytic activity^22^, the adsorption energies of reactive intermediates can be tuned, in principle, by tailoring the geometric and electronic structures of material, resulting in the enhancement of activity^23^. However, modulations of the local structure at the atomic level to tune WO_3_ into an active HER catalyst still remains as a great challenge.

Here we present a facile thermal treatment to activate a commercial product of WO_3_ into a highly competitive earth abundant catalyst, the dark blue tungsten oxide (WO_2.9_), for electrocatalysing HER in acidic water (see Fig. 1 for schematic mechanism). Experimentally observed results demonstrate that the WO_2.9_ with tailored structure exhibits excellent HER activity with a small overpotential of −70 mV at the current density of 10 mA cm^−2^ and a Tafel slope of 50 mV per decade. Further theoretical calculations indicate that its electrocatalytic capacity could be attributed to the modest binding energy with adsorbed atomic hydrogen. The findings in this work may hold the promise for the development of more practical non-Pt catalysts for electrocatalytic hydrogen evolution and other scalable technologies that harness renewable energy and convert it to H_2_, for example, the proton exchange membrane electrolysis in acidic environment.

## Results

### Electron microscopy

**Figure 2:**
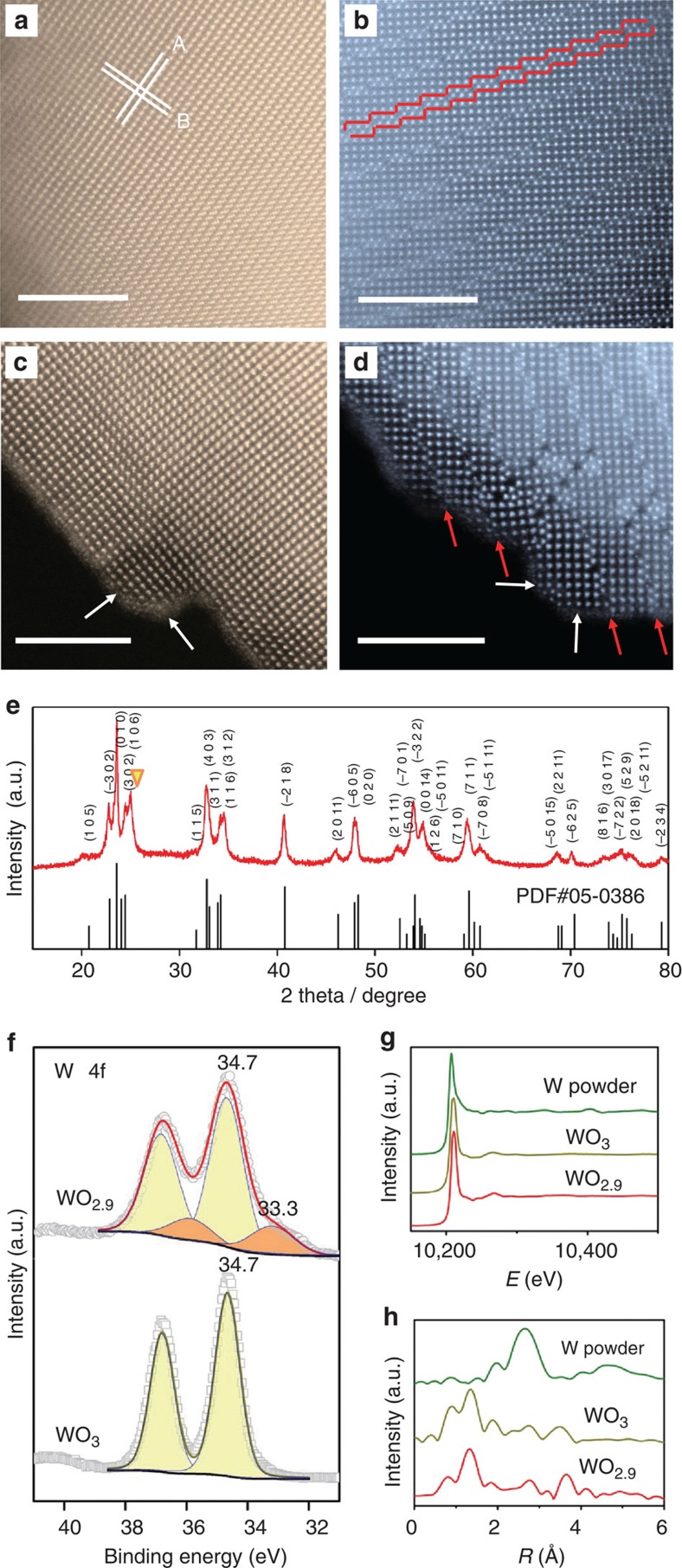
Structure analyses of WO_3_ and WO_2.9_ samples. High-angle annular dark-field scanning transmission electron micrograph (HAADF-STEM) images of the samples WO_3_ (**a**,**c**) and WO_2.9_ (**b**,**d**). W atoms can be clearly observed, yellow spots in **a**,**c** and blue spots in **b**,**d**. The bulk structures for (**a**) WO_3_, showing continuous lattice fringes (white lines, A for [002] and B for [200]) and (**b**) WO_2.9_, revealing a regularly stair-step shape (red lines). The typical surface structures for (**c**) WO_3_ and (**d**) WO_2.9_. White and red arrows point the surface structure that is similar to bulk structure of WO_3_ and WO_2.9_, respectively. Scale bar, 5 nm. (**e**) X-ray diffraction pattern of sample WO_2.9_ we synthesized in this work, which is in good agreement with the calculated diffraction pattern of bulk WO_2.9_ with an extra peak (inverted triangle). theta, diffraction angle. (**f**) X-ray photoelectron spectroscopy spectra showing the W 4*f* core level peak region of the samples WO_2.9_ and WO_3_. (**g**) The normalized X-ray absorption near-edge structure spectra at the W L_3_-edge of the metallic W powder, WO_3_ powder and WO_2.9_ catalyst. (**h**) The k^3^-weighted Fourier transform spectra from extended X-ray absorption fine structure.

To prepare the WO_2.9_ electrocatalyst, commercially available WO_3_ powder was well grinded and thermally treated in a reduction atmosphere, which would readily modulate the local atomic structure of WO_3_ (see more details in Methods). The colour of the sample changes from light yellow (commercial product of WO_3_) to dark blue after the modifications (Supplementary Fig. 1). Scanning electron microscope (SEM) images reveal the as-prepared WO_2.9_ nanoparticles with a mean diameter of 100 nm, which exhibits negligible difference comparing to the WO_3_ sample (Supplementary Fig. 2), indicating that the modification process in this work may not result in the aggregation of the nanoparticles. Moreover, the energy-dispersive spectrometer mapping, together with corresponding SEM image (Supplementary Fig. 3), clearly shows the existence of the elemental W and O in the WO_2.9_ sample. To further reveal its structure, a JEM-ARM200F scanning transmission electron microscopy (STEM) fitted with a double aberration corrector for both probe-forming and the imaging lenses is used to perform high-angle annular dark-field (HAADF) imaging. As the contrast exhibits an approximately Z^1.7^ dependency for HAADF imaging, the arrangement of crystallographic structure after modification can be identified directly at the atomic scale. Figure 2 presents the HAADF images of WO_3_ and WO_2.9_ samples, where W atoms are clearly observed (yellow spots for WO_3_ and blue spots for WO_2.9_). As shown in Fig. 2a, WO_3_ displays continuous lattice fringes with lattice spacing of 0.382 and 0.366 nm corresponding to the (002) and (200) atomic planes, respectively (white lines, marked as A and B), whereas WO_2.9_ exhibits an extended and ordered defect structure (Magnéli phase) with a regularly stair-step shape intermittently (red lines in Fig. 2b)^24^. Specifically, the bulk atomic structure of either WO_3_ or WO_2.9_ sample can also be extended to the surface, indicating the similar geometrical structure between bulk and surface (Fig. 2c,d, marked by white and red arrows). We emphasize that the surfaces and main parts of the local structures between WO_3_ and WO_2.9_ are similar, except for the stair-step shape lattice fringes in WO_2.9_. This suggests that the tailored electronic structure of the stair-case-shaped lattice fringes would be responsible for the enhanced HER performance of WO_2.9_.

### X-ray analyses

In addition to the HAADF-STEM study, Fig. 2e displays the X-ray diffraction (XRD) pattern of the as-synthesized WO_2.9_ electrocatalyst. The XRD pattern of the WO_2.9_ sample contains an extra peak that might belong to the WO_2.83_(−404) face (JCPDS Card No. 36-0103), and the other peaks are assigned well to monoclinic WO_2.9_ bulk (JCPDS Card No. 05-0386). On the other hand, the XRD pattern of WO_3_ sample illustrates the pure WO_3_ phase (Supplementary Fig. 4, JCPDS No. 43-1035). Besides, the characteristic peaks in Raman spectrum of the sample WO_2.9_ are broad and weak compared with those of sample WO_3_ (see details in Supplementary Fig. 5 and Supplementary Table 1), which could be attributed to the local lattice imperfections^25^, revealing the absence of partial O atoms in WO_2.9_ sample. Figure 2f reports our surface analysis for both WO_2.9_ and WO_3_ samples with the X-ray photoelectron spectroscopy (XPS) technique. For the WO_2.9_ sample, two major tungsten species, W^6+^ (4*f*_7/2_=34.7 eV) and W^5+^ (4*f*_7/2_=33.3 eV), are found on its surface, showing the existence of W^5+^ (ref 26). On the other hand, deconvoluted W 4*f* doublet peaks of the WO_3_ sample suggest that tungsten is solely in the state of W^6+^ (W 4*f*_7/2_=34.7 eV)^27^. Moreover, the peaks in the XPS survey scans of the materials before and after the modulations can be only assigned to the W, O and C elements (Supplementary Fig. 6), indicating the inexistence of other elements^28^. To know the neighbours of the W atoms, the WO_2.9_ sample was thus characterized by means of the W L_3_-edge X-ray absorption fine structure (XAFS). The W L_3_-edge white line derives from electron transitions from the 2*p*_3/2_ state to a vacant 5*d* state, and Fig. 2g presents the W L_3_-edge X-ray absorption near-edge structure spectra of the WO_2.9_ sample and the reference samples. The Fourier-transformed spectra of W L_3_-edge extended XAFS of the samples are shown in Fig. 2h. The peaks in the range 1–2 Å and around 3 Å appear in the curves of bulk WO_3_ and metallic W samples, respectively, owing to the W–O shell and W–W shell. Thus, the only peak in WO_2.9_ sample at 1–2 Å is believed to be the contribution from W–O binding, indicating the absence of metallic W–W bond, which is consistent with the results of XPS W 4*f* region that the metallic tungsten species (W^0^, 4*f*_7/2_=30.0 eV) could be hardly detected (Supplementary Fig. 7). On the basis of these results, the tungsten species in the as-synthesized catalyst could be the WO_2.9_ phase with only W–O bond.

### Electrochemical hydrogen evolution reaction

**Figure 3:**
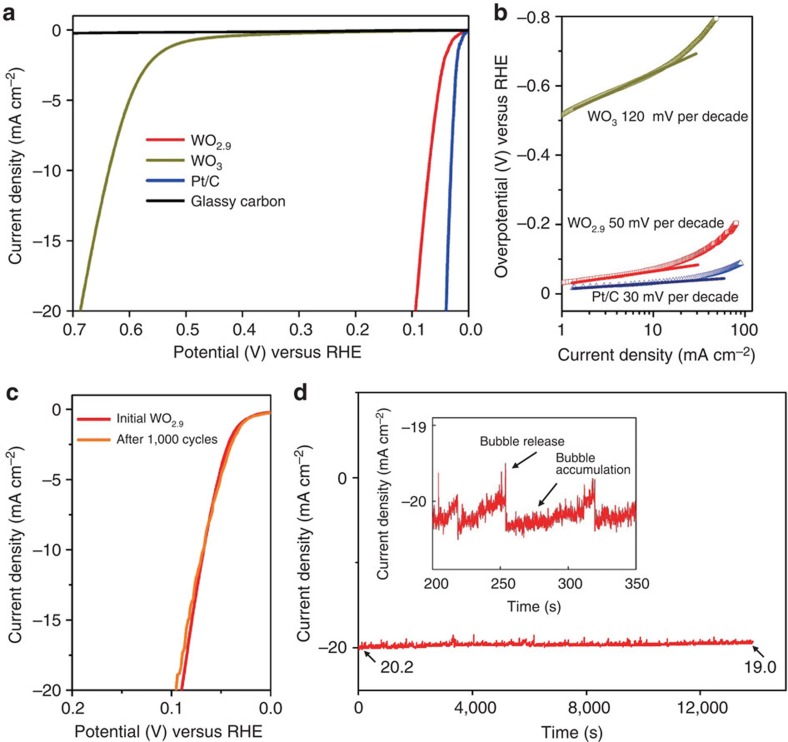
Hydrogen evolution reaction electrocatalytic properties. (**a**) Polarization data for the WO_2.9_ electrodes, along with WO_3_, Pt/C and glassy carbon for comparison. (**b**) Tafel plots of the polarization curves of the WO_2.9_, WO_3_ and Pt/C. (**c**) Polarization data for WO_2.9_ sample sweeps between −0.3 and +0.1 V versus RHE, showing negligible current density loss even after 1,000 CV cycles. (**d**) Time dependence of current density under static overpotential of −0.1 V. Inset is an enlargement of an area in **d**. All electrochemical studies were performed in 0.5 M H_2_SO_4_ at room temperature.

The electrodes for HER were prepared by drop casting a fixed volume and concentration of catalysts from an aqueous suspension onto glassy carbon disc (see more details in Methods). The HER with WO_2.9_ nanoparticles as the catalyst on glassy carbon electrode (GCE) was measured using a standard three-electrode electrochemical configuration in 0.5 M H_2_SO_4_ electrolyte deaerated with hydrogen. The electrodes were prepared by depositing approximately one continuous layer of WO_2.9_ sample over the electrode surface area. The polarization curves (not *iR* corrected) showing the normalized current density versus voltage (*j* versus V) for the WO_2.9_ catalyst along with commercial Pt/C (5%) and commercial WO_3_ powder for comparison, are shown in Fig. 3a. Compared with blank glassy carbon, the electrode coated with bulk WO_3_ exhibits a poor overpotential (*η*) value of −637 mV at the current density of 10 mA cm^−2^, demonstrating the electrocatalytically inactive for proton reduction kinetics of the commercial WO_3_ powder. In contrast, WO_2.9_ catalyst exhibits a small *η* value of −70 mV at the current density of 10 mA cm^−2^, indicating that the tailored structure effectively reduces the energy input for activating the HER. Moreover, for driving a current density of 20 mA cm^−2^, WO_2.9_ electrocatalyst only requires an overpotential of −94 mV (not *iR* corrected), indicating a performance evidently exceeding most of the reported noble-metal-free HER catalysts (see details in Supplementary Table 2). These results imply that fast electron transfer and HER activation occur on the WO_2.9_ electrocatalyst (Supplementary Movie 1). Further, the linear portions of the Tafel plots (Fig. 3b) were fit to the Tafel equation (*η*=*b* log *j*+*a*, where *j* is the current density and *b* is the Tafel slope)^29^, yielding Tafel slopes of ∼30, ∼50 and ∼120 mV per decade for Pt/C, WO_2.9_ and WO_3_ samples, respectively. The turnover frequencies (TOFs) were estimated for the *η* value of −100 and −200 mV using both theoretical and experimental surface areas for the HER in 0.50 M H_2_SO_4_ (Supplementary Note 1)^13^,^30^. The surface area of the WO_2.9_ catalyst is about 48.3 m^2^ g^−1^ determined by Brunauer–Emmett–Teller study, and the TOFs (per surface W atom) were calculated to be 8.04 s^−1^ at −100 mV and 24.76 s^−1^ at −200 mV. Theoretical TOF values, estimated geometrically by assuming 100-nm spherical particles of WO_2.9_, can be 4.64 s^−1^ at −100 mV and 14.29 s^−1^ at −200 mV. In addition, the HER inherent activity of these catalysts was evaluated by the exchange current density (*j*_0_). The *j*_*0*_ of WO_2.9_ catalyst is 0.40 mA cm^−2^ with a surface area of 0.97 cm^2^ on the working electrode (0.02 mg loading), which outperforms the value of 5.0 × 10^−5^ mA cm^−2^ for bulk WO_3_ (Table 1) and can be superior to those for other reported nonprecious HER catalysts (Supplementary Table 2). The high electrode kinetic metrics (including the overpotential of −70 mV at the current density of 10 mA cm^−2^ and the Tafel slope of 50 mV per decade) and large *j*_0_ (only half lower than the value of 0.93 mA cm^−2^ for Pt) highlight the exceptional H_2_ evolving efficiency of the WO_2.9_ catalyst.

Cyclic voltammetry (CV) was swept between −0.3 and +0.1 V (versus the reversible hydrogen electrode potential, RHE) were applied to the WO_2.9_-decorated working electrodes (Fig. 3c). After 1,000 CV sweeps, the overpotential required to achieve current densities of 10 mA cm^−2^ shows negligible change (from 70 to 71 mV), which remains higher than those of the benchmark catalysts (Supplementary Table 2). Moreover, we swept the CV towards positive potential up to +1.0 V (versus RHE) with scan rate of 0.02 V s^−1^ for 50 times (Supplementary Fig. 8). However, the WO_2.9_ catalyst shows an undesirable degradation of HER performance, indicating that it could hardly withstand excursions to positive potentials. Continuous HER at a static overpotential was also conducted. As shown in Fig. 3d, when an overpotential of −0.1 V was applied, a continuous HER process occurred to generate molecular H_2_. The as-measured time-dependent curve is in typical serrate shape, which could be attributed to the alternate processes of bubble accumulation and bubble release (inset in Fig. 3d). The amount of the decay of the WO_2.9_ catalyst is about 5.9% current loss after 14,000 s, which might be owing to the partial detachment of the catalyst caused by the continues bubbles releasing or the remaining of H_2_ bubbles on the surface of the electrode that hindered the reaction. The current density levelled out at an average of 19.6 mA cm^−2^ with the WO_2.9_ working electrode (0.07 cm^2^ surface area, 0.285 mg cm^−2^ loading), resulting in passage of 19.2 C of charge. On the other hand, control experiments run under identical conditions, but with the WO_3_ sample and without the catalyst, both showed no current. We further established the HER scale after the static overpotential test of the WO_2.9_ catalyst via a gas chromatograph (GC-2014C) with the argon as carrier gas. The total H_2_ amount is about 95 μmol, which is consistent with the theoretical value of 99.5 μmol by assuming that every electron is used for the reduction of protons.

In addition, we performed the XRD and XPS techniques to determine the structure of the WO_2.9_ catalyst after these electrocatalytic tests. As shown in Supplementary Fig. 9, diffraction peaks in pattern of sample WO_2.9_ remains at the similar intensity and position compared to those in Fig. 2e, revealing the unchanged local structure of WO_2.9_ catalyst. Moreover, the deconvoluted W 4*f* doublet peaks of the catalyst after the tests exhibit negligible difference. The dispersion of W^5+^ in both samples, which can be evaluated by the relative XPS intensity ratio of W^5+^ atom to W^6+^ atom, shows negligible change, remaining as 0.182 for the before and after samples. Specifically, we also detected the Pt 4*f* core level peak region to check for the possible impurities, and the existence of Pt can be thus safely ruled out. All results suggest that the origin of the excellent HER capacity could be attributed to the tailored electronic structure of WO_2.9_ catalyst by means of local atomic structure modulations.

### Density functional theory studies

**Figure 4:**
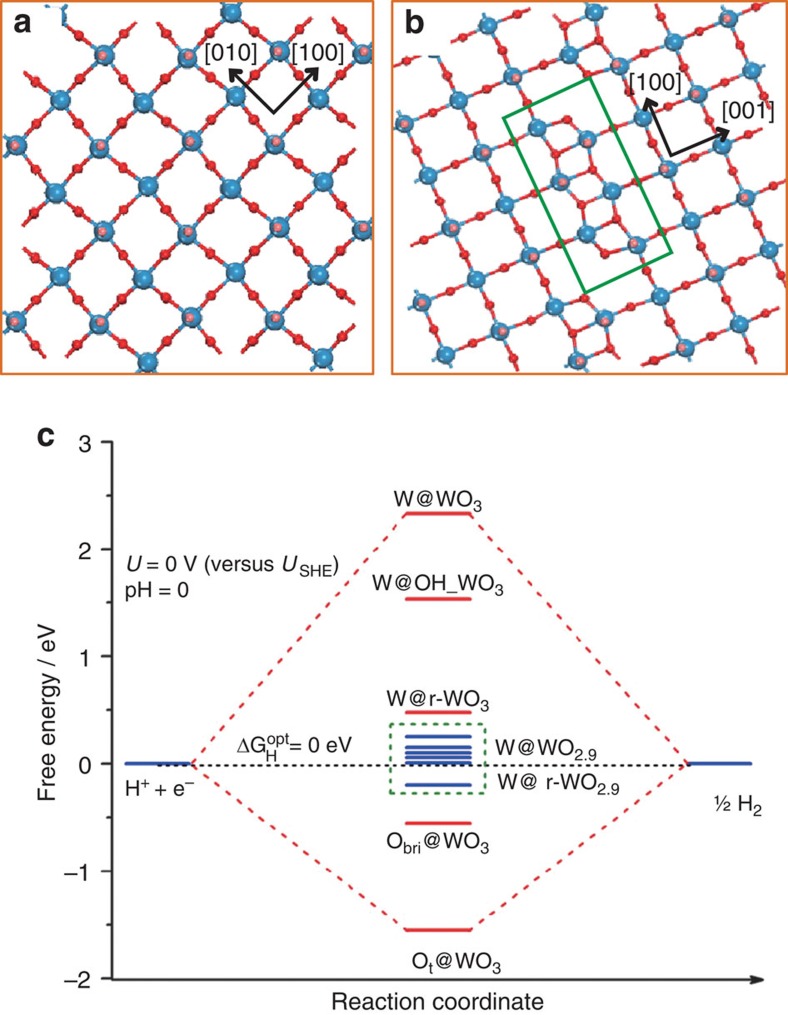
Density functional theory calculations. Top views of the optimized configuration of monoclinic WO_3_(001) (**a**) and WO_2.9_(010) surface (**b**), in which the box indicates the new characteristic configuration possessed by WO_2.9_ relative to WO_3_. Blue balls represent W atom, red for O and pink for the surface terminal O. This notation is used throughout this work. (**c**) Calculated free energy diagram of HER at the equilibrium potential (*U*=*U*_SHE_) for a series of active sites on WO_3_(001) and WO_2.9_(010).

On the basis of the above experimental investigations, we thus systematically examined the binding ability of their respective most stable surface, that is, WO_2.9_(010) and WO_3_(001) by virtue of extensive first-principle density functional theory (DFT) calculations (see details in Fig. 4, Supplementary Figs 10–13 and Supplementary Note 2). The calculated parameters (Supplementary Table 3) show that the adsorption energy on WO_2.9_(010) is largely enhanced relative to that on WO_3_(001). For example, the adsorption energy can be −0.19 eV on WO_2.9_(010), and accordingly, the free energy change of the discharge step (H^+^+e^−^→H*) for HER at the standard condition (*U*=0 V versus *U*_SHE_, pH=0) can be calculated to be 0.01 eV, fulfilling the Δ*G*_H_=0 eV requirement, and thus its high catalytic activity could be expected. Moreover, the formed terminal OH could further adsorb H and form H_2_O, resulting in the possible reduction. We thus considered the surface reduction by removing all the terminal O from the *p*(1 × 1) WO_2.9_(010) slab, corresponding to a W/O ratio of W_60_O_154_, whose adsorption energy can be further improved by the order of only ∼0.30 eV compared with the clean WO_2.9_(010) surface (Supplementary Table 4). From Fig. 4c, the activity can remain at a high level, despite being a little lower to some extent relative to clean WO_2.9_(010). Therefore, it could be rationalized that WO_2.9_ exhibits a high and stable activity. We further performed a brief electronic analysis to provide insight into the enhanced H adsorption strength of WO_2.9_(010). One can see that the highest occupied *d*-orbital of surface W_5c_ largely affects the binding ability toward H atom (Supplementary Figs 14–16), and the appearance of *d*-band around the Fermi level for WO_2.9_(010) would be an important factor for the strengthened binding ability compared with WO_3_. In addition, the calculated work function suggests that WO_2.9_ has a higher Fermi level than WO_3_ by 0.70 eV, which may facilitate the reduction process to occur kinetically.

### Stability of WO_2.9_ electrocatalyst

To further probe the stability of the WO_2.9_ catalyst during electrocatalytic hydrogen evolution in 0.5 M H_2_SO_4_, the CV of WO_2.9_ catalyst was swept between −0.3 and +0.1 V for 10,000 times (Supplementary Fig. 17). As with many metal oxides, WO_2.9_ nanoparticles also suffer from the undesired structure change in acidic water for a long time; they are slightly soluble after these additional accelerated degradation studies, which results in the degradation of electrocatalytic activity. The overpotential increased from −94 to −162 mV at the current density of 20 mA cm^−2^ after 10,000 CV sweeps. XRD pattern in Supplementary Fig. 18 reveals partial formation of WO_2.8_ phase in catalyst, and the XPS spectrum also shows an enhancement of the W^5+^ doublet peaks in the spectrum from those of fresh WO_2.9_ sample (Supplementary Fig. 19), indicating the high oxygen vacancies of the atomic structure in acidic water for the rigorous tests. It should be noted, however, that the WO_2.9_ catalyst is still much more thermodynamically stable in acidic water than most metal oxides. Further investigation is needed to obtain a clear picture of the exact microscopic changes to their surface chemistry and lattice structure. We believe that, with further research, the deactivation may be reduced or eliminated, for example, by integrating WO_2.9_ nanoparticles with a graphene shell or other nanostructures^31^,^32^,^33^.

## Discussion

The WO_2.9_ electrocatalyst prepared in this work exhibits excellent HER activity with a very low cathodic overpotential of −70 mV at the current density of 10 mA cm^−2^ and a small Tafel slope of about 50 mV per decade. By modulating the local atomic structure of WO_3_ at the atomic scale, an extended and ordered defect structure (Magnéli phase) is formed, resulting in preferentially exposed W sites with modified electronic structure that show a greatly enhanced catalytic activity for hydrogen evolution. We anticipate the transition metal oxide materials with suitable metal hydrogen binding energy may also hold the promises to compete against the best precious metal catalysts available for HER, compared with the well-studied carbides, nitrides, phosphides and sulfides.

## Methods

### Synthesis of catalyst

In the preparation of the material, a two-step synthesis process was involved. First, 1 g of commercial WO_3_ (of analytically pure grade, 99.9%, Sinopharm) was carefully ground, which was carried out in a ball mill with the wet grinding method (ethanol, 24 h under rotation speed of 300 r.p.m.). Then, we prepared the thermally treated samples through annealing ground WO_3_ in hydrogen atmosphere (1 bar, 10% H_2_, 90% Ar, 100 s.c.c.m. flow) in a tube furnace at 500 °C for 60 min. The resulting powder can be collected after the tube furnace cooling down to room temperature.

### Electrochemical measurements

Four microgram of catalyst and 80 μl of 5 wt% Nafion solution (Sigma-Aldrich) were dispersed in 1 ml of 4:1 v/v water/ethanol by at least 30-min sonication to form a homogeneous ink. Then, 5 μl of the catalyst ink (containing 20 μg of catalyst) was loaded onto a GCE of 3 mm in diameter (loading 0.285 mg cm^−2^). The area of coated electrodes may exceed that of the glassy carbon disc, but we calculate all the current densities using the geometric value. The WO_2.9_ modified GCE was left to dry at 40 °C. For comparison, GCEs were also modified with commercial WO_3_ from Sinopharm (99.9%) and Pt/C (5%) from Alfa Aesar.

All electrochemical studies were performed using a CHI 660 potentiostat (CH Instruments, China) in a three-electrode setup with a modified glassy carbon working electrode, an Ag/AgCl/KCl (3.5 M) electrode as a reference, a graphite rod (spectral purity, 3-mm diameter) as a counter electrode and deaerated with hydrogen before use. The electrocatalytic activity of WO_2.9_ towards HER was examined by polarization curves using linear sweep voltammetry at a scan rate of 5 mV s^−1^ in 0.5 M H_2_SO_4_ at room temperature. All of the potentials in this work were calibrated to a RHE. The amount of evolved H_2_ was monitored by a gas chromatograph (GC-2014C) with argon as carrier gas.

### Catalysts characterization

The crystal structure was determined using XRD (D/MAX 2550 VB/PC) and Raman spectroscopy (Renishaw, inVia+Reflex). The structure of the catalysts was examined by SEM (S-3400N) and TEM (TECNAI F-30, 300 kV). Further, the chemical states of the elements in catalysts were studied by XPS (ESCALAB 250Xi), and the binding energy of the C 1 s peak at 283.9 eV was taken as an internal reference. W L_3_-edge absorption spectra (extended XAFS) were performed on the 1W1B beamline of the Beijing Synchrotron Radiation Facility, China, operated at ∼200 mA and ∼2.5 GeV. W foil and WO_3_ powder were used as the reference samples. All samples were measured in the transmission mode. Brunauer–Emmett–Teller surface area measurement was performed at 77 K on a Micromeritics ASAS 2460 adsorption analyzer in N_2_ adsorption mode.

### Theoretical calculation

All the spin-polarized calculations were performed with Perdew–Burke–Ernzerhof functional within the generalized gradient approximation using the Vienna Ab-initio Simulation Package (VASP) code, unless otherwise specified. The project-augmented wave method was used to represent the core-valence electron interaction. The valence electronic states were expanded in plane wave basis sets with energy cutoff at 450 eV. The occupancy of the one-electron states was calculated using the Gaussian smearing (SIGMA=0.05 eV). The ionic degrees of freedom were relaxed using the Broyden-Fletcher-Goldfarb-Shanno (BFGS) minimization scheme until the Hellman–Feynman forces on each ion were <0.05 eV Å^−1^. The transition states were searched using a constrained optimization scheme, and were verified when (i) all forces on atoms vanish; and (ii) the total energy is a maximum along the reaction coordination but a minimum with respect to the rest of the degrees of freedom.

To model the monoclinic WO_3_(001) surface, a nine-layer *c*( √2 × √2)R45° slab (10.772 × 10.805 Å^2^) with a vacuum layer of 15 Å was adopted, corresponding to (WO_3_)_32_ (128 atoms). For WO_2.9_(010) surface, an enough large seven-layer *p*(1 × 1) slab (23.839 × 12.202 Å^2^) was used as the model. Because of the large size of the WO_2.9_(010) supercell, *k*-point sampling was restricted to the *Γ* point only. It is worth noting that, all the atomic layers in the optimization of WO_2.9_(010) were allowed to relax and the surfaces were constructed based on the pre-optimized bulk unit cell (see the optimized lattice constants of the monoclinic WO_3_ and WO_2.9_ in Supplementary Table 5).

### Free energy calculation method

To obtain the free energy of the each elementary step, when involving H^+^+e^–^, the standard hydrogen electrode (SHE) was used as the reference in standard Gibbs free energy calculation of HER. As derived in our previous work, Gibbs free energy change (Δ*G*) of each elementary step can be calculated as follows:


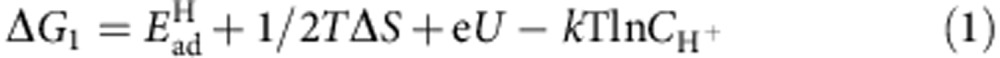



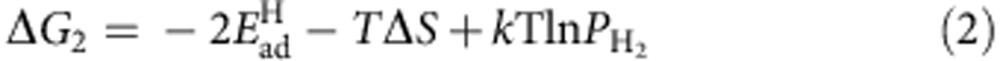


in which *U* is the electronic voltage versus SHE, while *P*_H_2__ and *C*_H^+^_ are the relative partial pressure of H_2_ and the relative concentration of H^+^ in the aqueous solution, respectively. At the standard condition, there are approximately Δ*G*_1_=*E*_ad_^H^+0.20 eV. The adsorption energy (*E*_ad_^H^) for hydrogen was obtained from the DFT calculation at 0 K relative to gas phase H_2_ molecule, which is defined as equation (3).


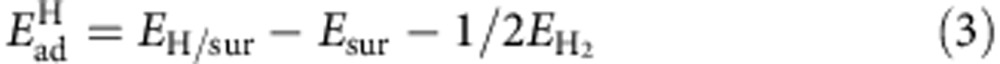


where *E*_H/sur_ and *E*_sur_ are the energy of the surface slab with and without atomic H adsorption, and *E*_H_2__ is the total energy of the H_2_ molecule in the gas phase. The more negative *E*_ad_^H^ is, the more strongly the species H binds on surface.

## Additional information

**How to cite this article:** Li, Y. H. *et al*. Local atomic structure modulations activate metal oxide as electrocatalyst for hydrogen evolution in acidic water. *Nat. Commun.* 6:8064 doi: 10.1038/ncomms9064 (2015).

## Supplementary Material

Supplementary Information Supplementary Figures 1-19, Supplementary Tables 1-5, Supplementary Notes 1-2 and Supplementary References.

Supplementary Movie 1 Hydrogen evolution reaction with WO_2.9_ nanoparticles as the catalyst on glassy carbon electrode was measured at overpotentials of -100, -200, -300 and -400 mV, using a standard three-electrode electrochemical configuration in 0.5 M H_2_SO_4_ electrolyte deaerated with hydrogen.

## Figures and Tables

**Figure 1:**
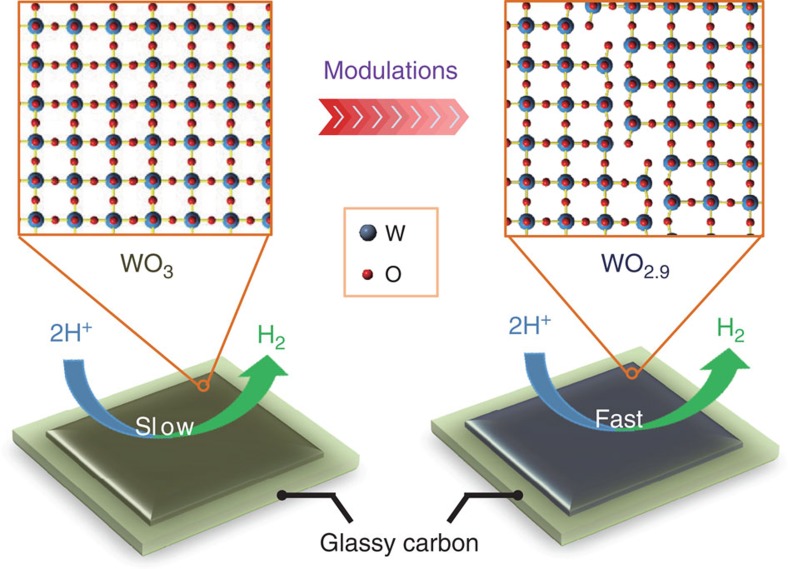
Plausible reaction mechanism of electrocatalytic H_2_ evolution. By means of local atomic structure modulations, the WO_2.9_ electrocatalyst with tailored electronic structure exhibits excellent HER activity, whereas the original WO_3_ sample is electrocatalytically inactive for proton reduction kinetics.

**Table 1:** Comparison of catalytic parameters of different HER catalysts.

**Catalyst**	**Current density (*****j*****, mA cm**^**−2**^)	**Corresponding overpotential (*****η*****, mV)**	**Tafel slope (mV per decade)**	**Exchange current density (*****j***_**0**_**, mA cm**^**−2**^)*
WO_3_	10	−637	120	5.0 × 10^−5^
WO_2.9_	10	−70	50	0.40
Pt/C†	10	−31	30	0.93

HER, hydrogen evolution reaction; RHE, reversible hydrogen electrode.

^*^j_0_ was calculated from Tafel curves using extrapolation method.

^†^Johnson–Matthey, 20 wt% Pt/XC-72.
