# Supplementary material for: Local atomic structure modulations activate metal oxide as electrocatalyst for hydrogen evolution in acidic water
Source: Nat Commun. 2015 Aug 19;6:8064. doi: 10.1038/ncomms9064 (PMC4560788; doi:10.1038/ncomms9064)
Supplement: Supplementary Information — Supplementary Figures 1-19, Supplementary Tables 1-5, Supplementary Notes 1-2 and Supplementary References. [file ncomms9064-s1.pdf]

Supplementary Information

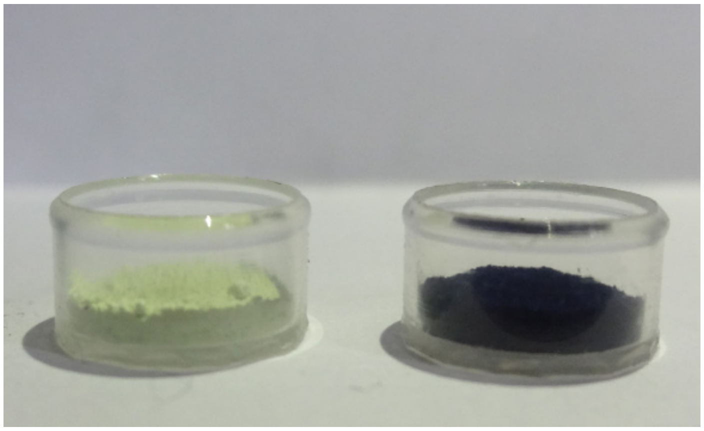

Supplementary Figure 1: A photo comparing the commercial product of WO_3 (light yellow) and thermally treated $WO_{2.9}$ electrocatalyst (dark blue).

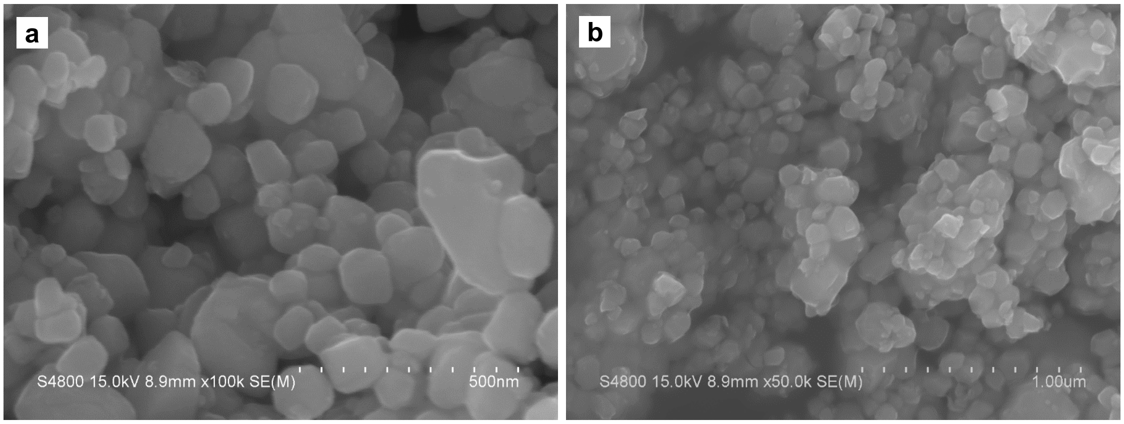

Supplementary Figure 2: SEM images of (a) $WO_{2.9}$ electrocatalyst and (b) WO_3 sample.

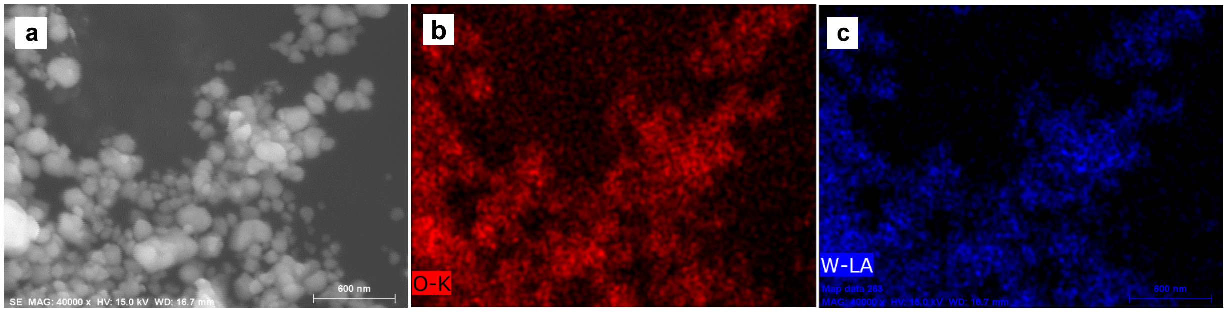

Supplementary Figure 3: (a) SEM image and elemental mappings of $WO_{2.9}$ sample: O (b) and W (c).

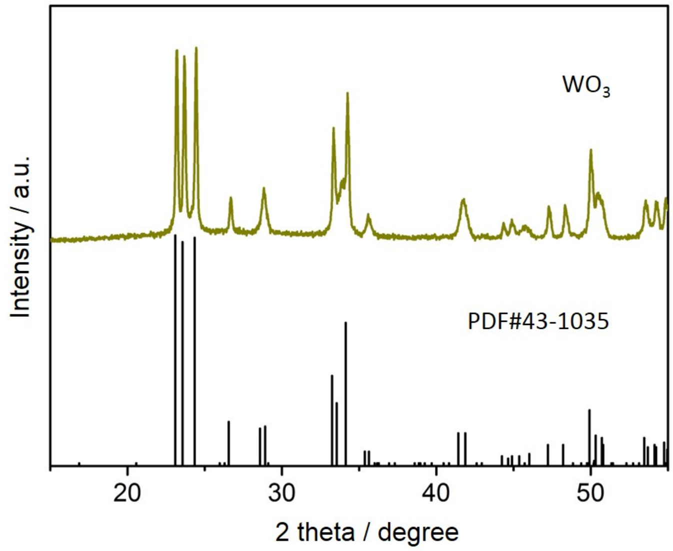

Supplementary Figure 4: XRD pattern of the commercial WO_3 sample, which can be in good agreement with the calculated diffraction pattern of bulk WO_3. theta, diffraction angle.

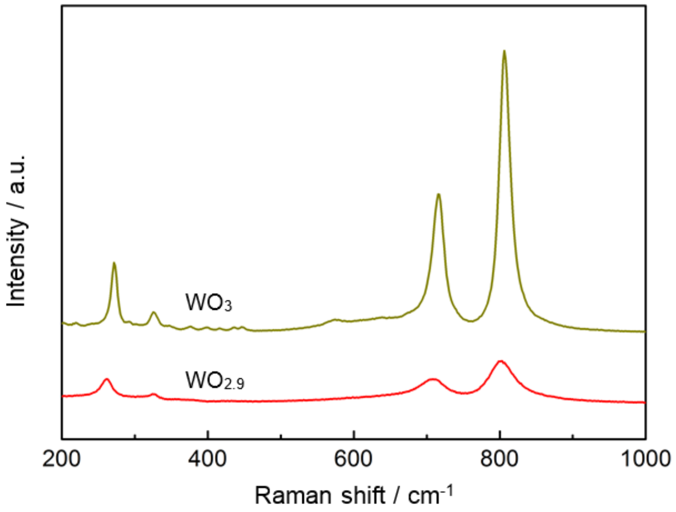

Supplementary Figure 5: Raman spectra of the $WO_{2.9}$ catalyst and commercial WO_3 sample (λ_{ex} = 514 nm). The $WO_{2.9}$ peaks are broader than those of WO_3. For example, the full width-at-half-maximum (FWHM) value of the peak around 800 cm^{-1} is 38.1 ± 0.8 cm^{-1} for the $WO_{2.9}$ sample, which is broader than that for WO_3 sample (17.8 ± 0.3 cm^{-1}).

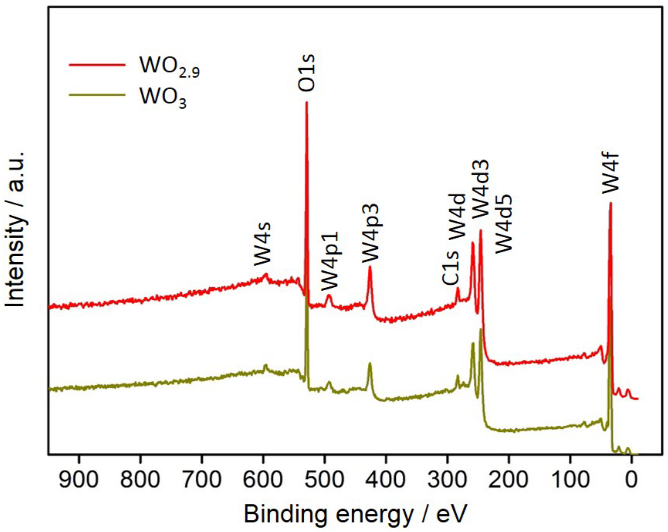

Supplementary Figure 6: XPS survey spectra of the $WO_{2.9}$ catalyst and commercial WO_3 sample.

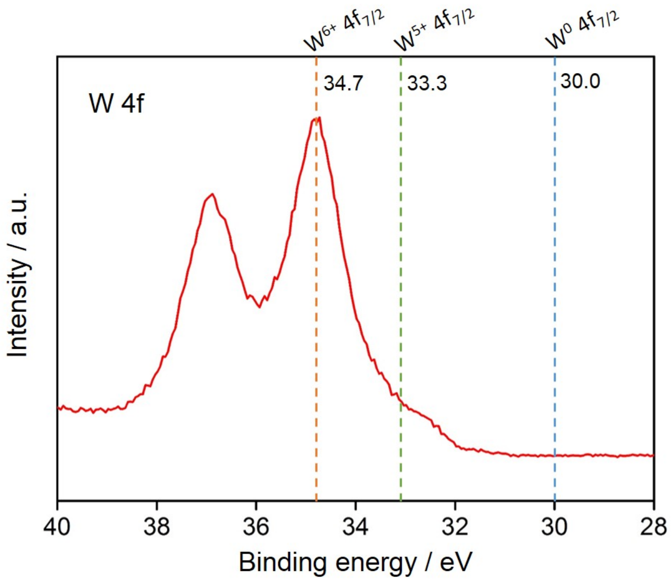

Supplementary Figure 7: X-ray photoelectron spectroscopy spectrum, showing the W 4f core level peak region of the $WO_{2.9}$ catalyst marked with tungsten in different valence states.

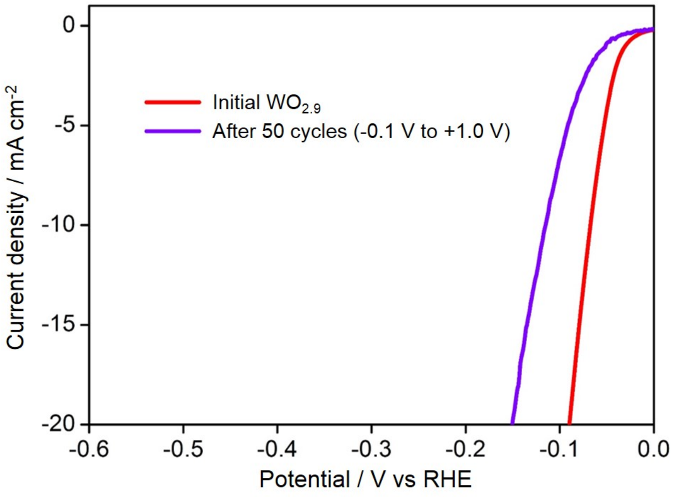

Supplementary Figure 8: Polarization data for $WO_{2.9}$ catalyst sweeps between -0.1 V and +1.0 V vs RHE, showing the current density changes after 50 CV cycles. Scan rate: 0.02 V s^{-1}.

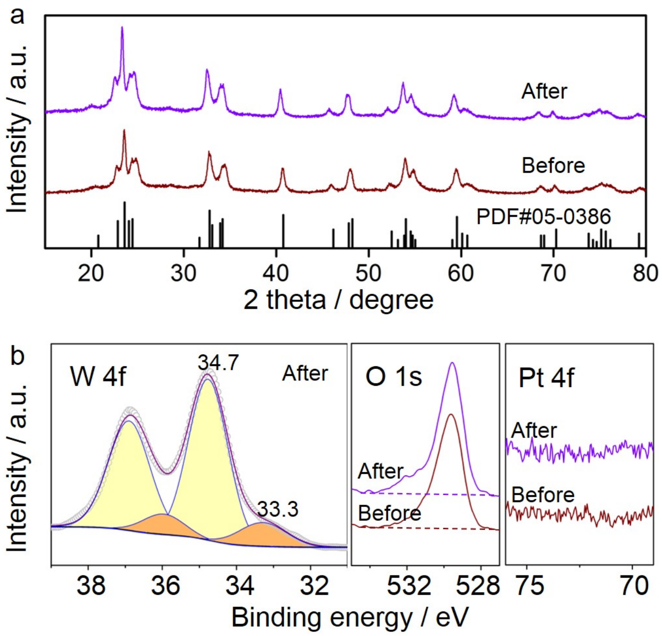

Supplementary Figure 9: X-ray analyses of the catalyst after electrocatalytic tests. (**a**) X-ray diffraction patterns of the $WO_{2.9}$ catalyst before and after the electrocatalytic tests, which are in good agreement with the calculated diffraction pattern of bulk $WO_{2.9}$. theta, diffraction angle. (**b**) XPS spectra of W 4f, O 1s, and Pt 4f for the $WO_{2.9}$ catalyst before and after the electrocatalytic tests. For Pt 4f region, the scan rate is 0.2 eV s^{-1} with an energy step size of 0.05 eV for 10 sweeps. For W 4f region, the scan rate is 1.0 eV s^{-1} with an energy step size of 0.05 eV for 1 sweep.

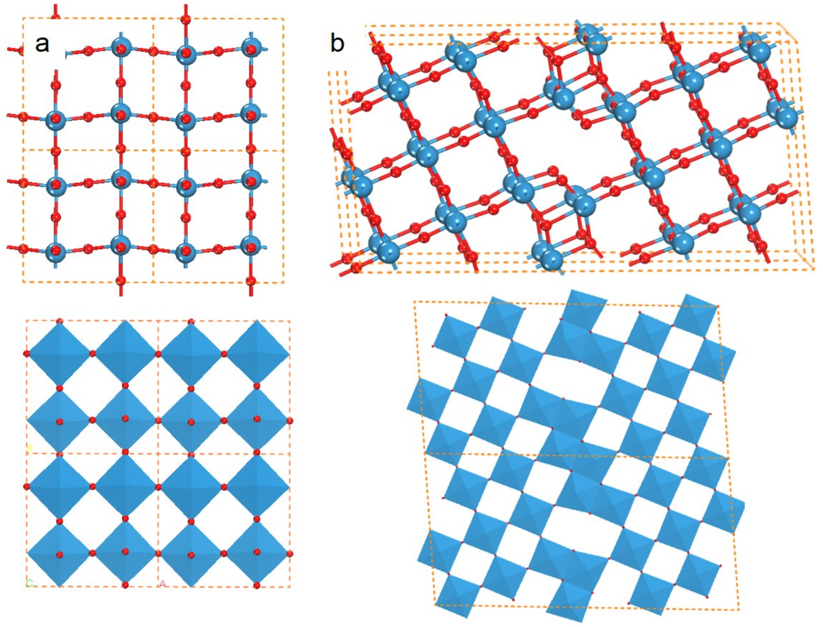

Supplementary Figure 10: Optimized bulk structures of monoclinic WO_3 (a), and monoclinic $WO_{2.9}$ (b), in which W and O atoms are represented in blue and red, respectively. These notations are used throughout in calculations in this work.

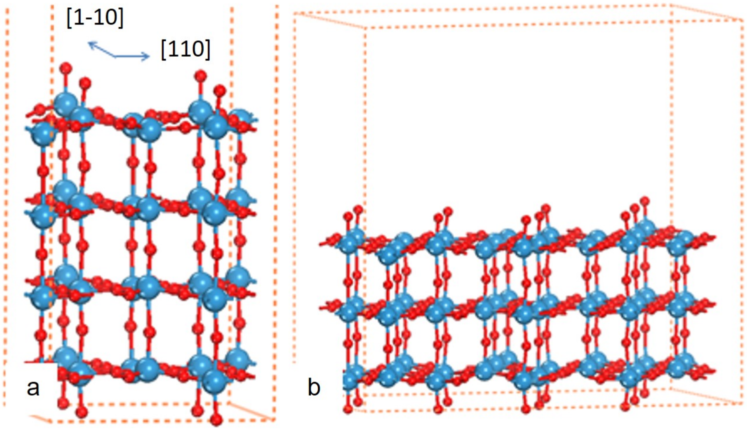

Supplementary Figure 11: (a) Side view of the reconstructed WO_3(001) by removing half the surface terminal O to the bottom layer to cancel the dipole, and (b) are side view of the stable termination configurations of $WO_{2.9}$(010) surface.

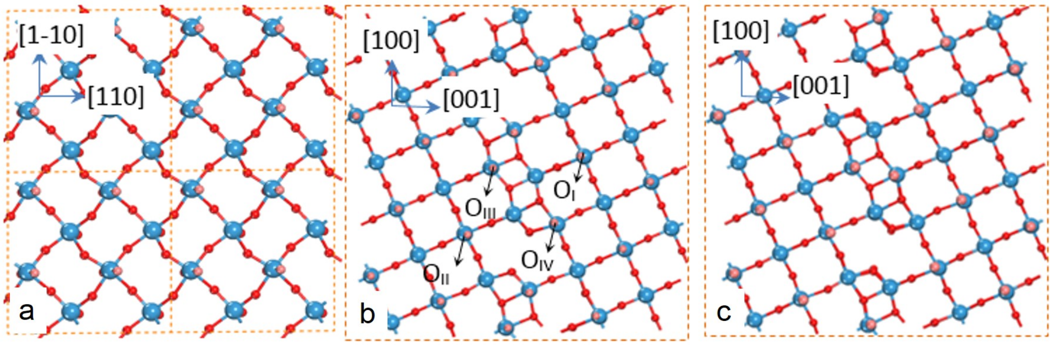

Supplementary Figure 12: (a) Top view of the optimized WO_3(001) surface; (b) and (c) are the two termination configurations of $WO_{2.9}$(010) surface, denoted as config_1 and config_2, respectively.

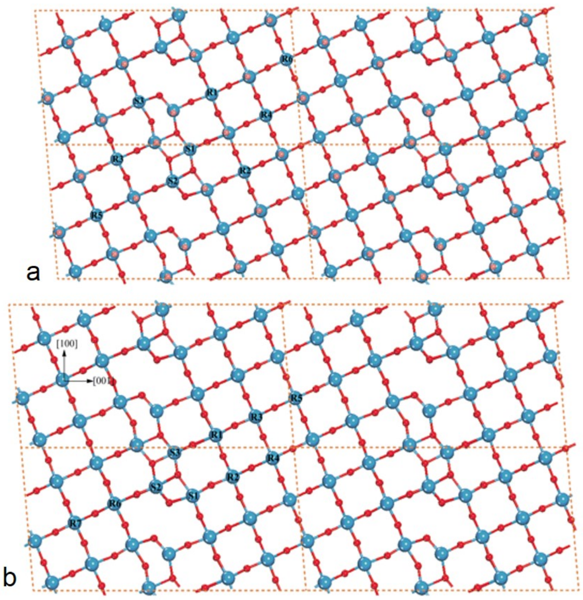

Supplementary Figure 13: Reprehensive W_{5c} adsorption site on $WO_{2.9}$(010) surface. (**a**) config_1; (**b**) reduced config_1.

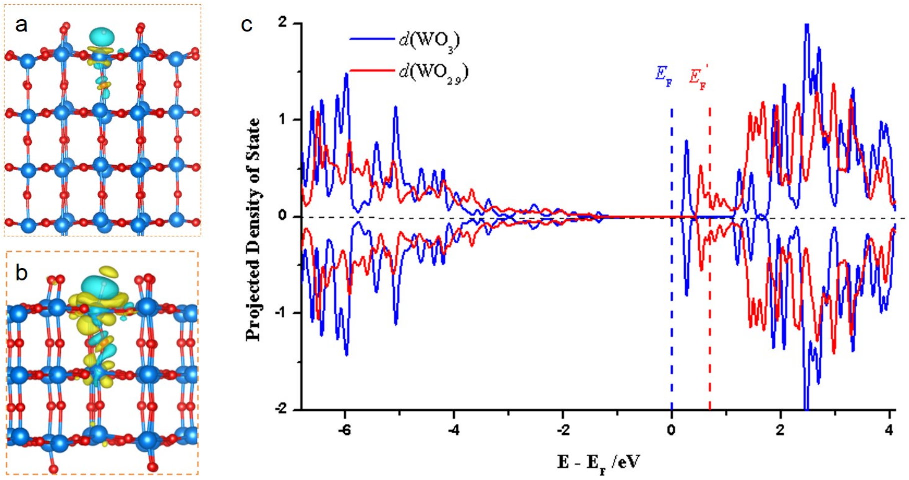

Supplementary Figure 14: Projected density of state on *d*-orbital of the surface W_{5c} atom on WO_3(001) and $WO_{2.9}$(010). (**a**) and (**b**) show the 3D contour plot of charge density difference for H adsorption at the W_{5c} site on WO_3(001) and $WO_{2.9}$(010) surface with the isovalue of 0.0015, in which light blue indicates the electronic accumulation and yellow for electronic depletion. (**c**) Projected density of state (PDOS) on *d*-orbital of the surface W_{5c} atom on WO_3(001) and $WO_{2.9}$(010), in which the Fermi energy level (E_F) of WO_3(001) is aligned to 0 eV, and E_F' gives the relative Fermi energy of $WO_{2.9}$(010) in alignment to the vacuum energy, indicating the higher Fermi level of $WO_{2.9}$(010) relative to WO_3(001) (E_F' - E_F = 0.70 eV).

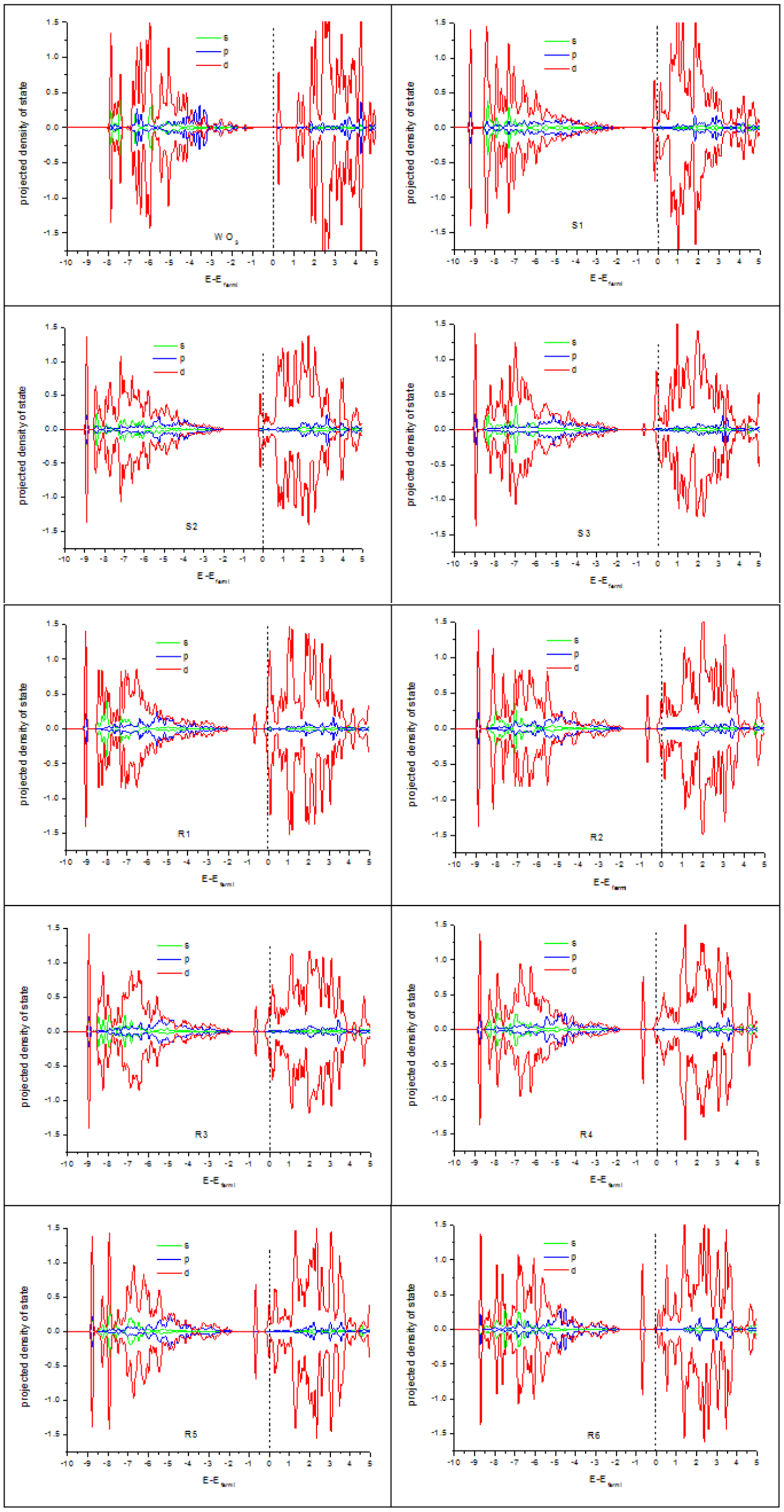

Supplementary Figure 15: Projected density of state (PDOS) of the surface W_{5c} atom on WO_3(001) and $WO_{2.9}$(010), in which the Fermi energy level (E_F) is aligned to 0 eV.

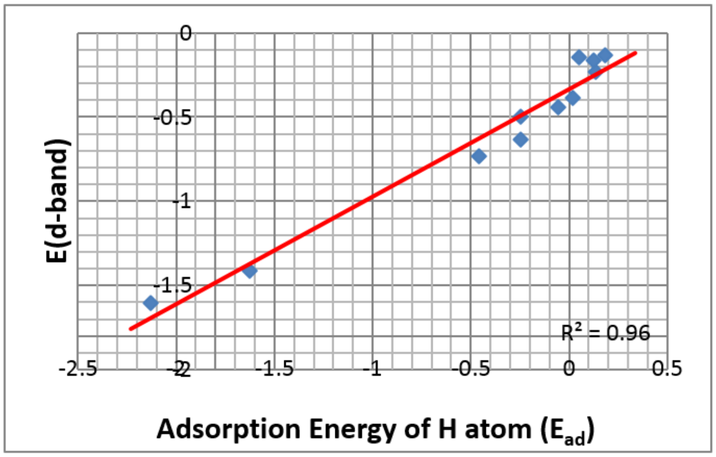

Supplementary Figure 16: The correlation between the H adsorption energy and the center of d band near the Fermi energy level (by integrating the range E_F-2 ~ E_F).

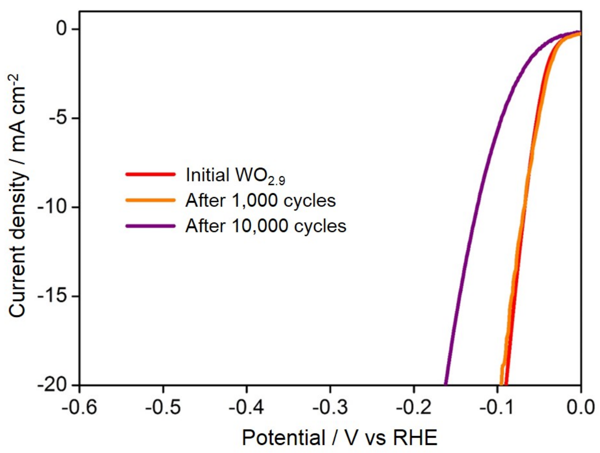

Supplementary Figure 17: Polarization data for $WO_{2.9}$ sample sweeps between -0.3 and +0.1 V vs RHE, showing the current density changes after 10000 CV cycles. Scan rate of 0.1 V s^{-1}.

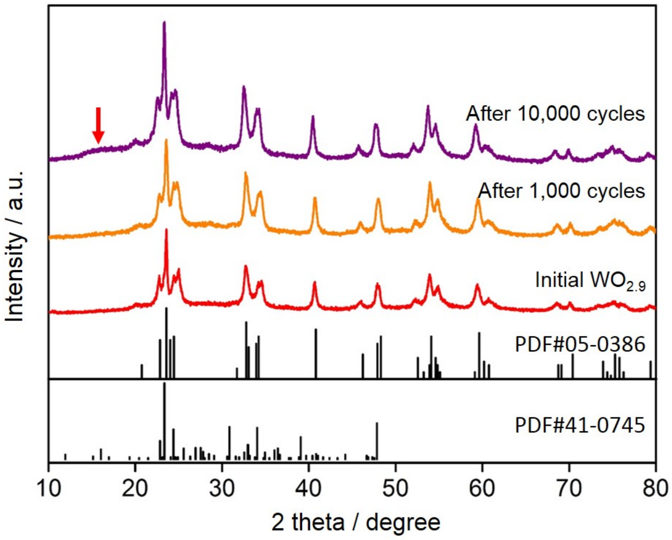

Supplementary Figure 18: X-ray diffraction patterns of the catalyst before and after 1000 and 10000 CV cycles. The calculated diffraction patterns of $WO_{2.9}$ (JCPDS Card No. 05-0386) and $WO_{2.8}$ (JCPDS Card No. 41-0745) phases are listed for comparison. theta, diffraction angle.

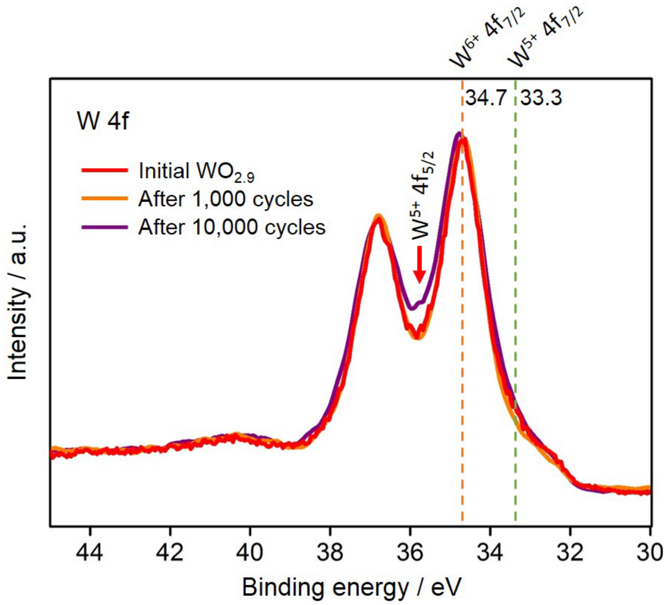

Supplementary Figure 19: X-ray photoelectron spectroscopy spectra showing the W 4f core level peak region of the $WO_{2.9}$ catalyst before and after 1000 and 10000 CV cycles.

Supplementary Table 1: The FWHM values (k) of the peaks in Raman spectra of $WO_{2.9}$ and WO_3 samples.

Notation in Supplementary Fig. 5	k / cm^{-1} $WO_{2.9}$	k / cm^{-1} WO_3
A	9.8 ± 0.5	5.9 ± 0.2
B	13.8 ± 0.4	9.1 ± 0.2
C	35.9 ± 0.9	17.5 ± 0.4
D	38.1 ± 0.8	17.8 ± 0.3

Supplementary Table 2: Comparison of selected state-of-art non-Pt HER electrocatalysts in acidic aqueous media.

Catalyst (mg cm^{-2})	Current density (j, mA cm^{-2})	Corresponding overpotential (η, mV)	Tafel slope (mV dec^{-1})	Exchange current density (j_0, mA cm^{-2})	Ref.
**FeP NPs/Ti (1.0)**	10	-50	37	0.43	***ACS. Nano.***, 2014, 8, 11101-11107
**FeP NA/Ti[a] (3.2)**	10	-55	38	0.42	***Angew. Chem. Int. Ed.***, 2014, 53, 12855-12859
**CoP/CC (0.92)**	10	-67	51	0.29	***J. Am. Chem. Soc.***, 2014, 136, 7587-7590
**$WO_{2.9}$ (0.285)**	**10**	**-70[b]**	**50[b]**	**0.40**	**this work**
**porous g-C_3N_4 with N-doped graphene sheets (0.57)**	10	-80	49.1	0.43	***ACS Nano.***, 2015, 9, 931-940
**phosphorus-modified WN/rGO (0.337)**	10	-85	54	0.35	***Adv. Funct. Mater.***, 2015, 25, 2693-2700
**MoS_x/NCNT (0.285)**	10	-110[b]	40[b]	3.31×10^{-2}	***Nano. Lett.***, 2014, 14, 1228-1233
**Ni_2P hollow NPs/Ti (1.0)**	10	-116	46	3.3×10^{-2}	***J. Am. Chem. Soc.***, 2013, 135, 9267-9270
**interconnected network of MoP NPs (0.36)**	10	-125	54	0.086	***Adv. Mater.***, 2014, 26, 5702-5707
**Mo_2C/CNT-graphene (0.65-0.67)**	10	-130	58	6.2×10^{-2}	***ACS. Nano.***, 2014, 8, 5164-5173
**$CoSe_2$ NP/CP[a] (2.5-3.0)**	10	-139	42.1	$(4.9 \pm 1.4) \times 10^{-3}$	***J. Am. Chem. Soc.***, 2014, 136, 4897-4900
**MoS_x/graphene/Ni foam[a] (5.01)**	10	-141	42.8	/	***Adv. Mater.***, 2013, 25, 756-760
**porous MoC_x nano-octahedrons (0.8)**	10	-142	53	0.023	***Nature Commun.***, 2015, 6, 6512
**CoS_2 NW/Graphite[a] (1.4-2.0)**	10	-145	51.6	2.8×10^{-3}	***J. Am. Chem. Soc.***, 2014, 136, 10053-10061
**MoS_2/RGO (0.285)**	10	-150	41	/	***J. Am. Chem. Soc.***, 2011, 133, 7296-7299
**Mo_2C/CNT (2.0)**	10	-152	55.2	1.4×10^{-2}	***Energy Environ. Sci.***, 2013, 6, 943-951
**porous C_3N_4 @ NG (0.57)**	10	-170	49.1	0.43	***ACS. Nano.***, 2015, 9, 931-940

**oxygen-incorporated MoS_2 NS (0.285)**	10	-180	55	1.26×10^{-2}	***J. Am. Chem. Soc.***, 2013, 135, 17881-17888
**exfoliated MoS_2 NS**	10	-195	54	/	***J. Am. Chem. Soc.***, 2013, 135, 10274-10277
**$Co_{0.6}Mo_{1.4}N_2$ (0.24)**	10	-200	/	0.23	***J. Am. Chem. Soc.***, 2013, 135, 19186-19192
**exfoliated WS_2 NS (0.1×10^{-3}-0.2×10^{-3})**	10	-240[b]	60	2.0×10^{-2}	***Nature Mater.***, 2013, 12, 850-855
**C_3N_4@NG (0.1)**	10	-240	51.5	3.5×10^{-4}	***Nature Common.***, 2014, 5, 3783
**Co-NRCNTs (0.28)**	10	-260	69	1.0×10^{-2}	***Angew. Chem. Int. Ed.***, 2014, 53, 4372-4376
**FeCo@NCNTs-NH (0.32)**	10	-270	74	/	***Energy Environ. Sci.***, 2014, 7, 1919-1923

[a] catalysts directly grown on the conductive substrate

[b] not *iR*-corrected

Supplementary Table 3: Calculated parameters of the WO_3(001) and $WO_{2.9}$(010) surface. It shows calculated H adsorption energies and bond length on various W_{5c} sites on WO_3(001) and $WO_{2.9}$(010) surface, as well as the corresponding Bader charges and *d*-band center near the Fermi level for the W_{5c} atom at the PBE level. In addition, the derived Gibbs free energy changes (ΔG_H) for the discharge step ($H^+ + e^- + * \rightarrow H^*$) at these W_{5c} site were also listed.

		E_{ad}(H)	ΔG_H	d(H-W)	Charge	$E_{d\text{-band}}$
WO_3(001)	W_{5c}	1.20	1.40	1.728	4.532	-1.606
	O_t	-1.42	-1.22	/	/	/
	O_{bri}	-0.70	-0.50	/	/	/
$WO_{2.9}$(010)	S1	-0.10	0.10	1.734	4.250	-0.160
	S2	-0.05	0.15	1.728	4.258	-0.146
	S3	-0.19	0.01	1.728	4.43	-0.129
	R1	-0.14	0.06	1.725	4.429	-0.227
	R2	0.05	0.25	1.726	4.412	-0.447
	R3	0.01	0.21	1.727	4.504	-0.388
	R4	0.25	0.45	1.727	4.375	-0.629
	R5	0.24	0.44	1.729	4.346	-0.499
	R6	0.46	0.66	1.730	4.408	-0.727

Supplementary Table 4: The calculated H adsorption energy at various W_{5c} sites on the reduced $WO_{2.9}$(010) surface corresponding to Supplementary Fig. 11b.

	S1	S2	S3	R1	R2	R3	R4	R5	R6	R7
E_{ad}(H) /eV	-0.31	-0.36	-0.44	-0.27	-0.20	-0.15	-0.14	-0.11	-0.30	-0.18

Supplementary Table 5: Structure information and the optimized lattice constants of WO_3 and $WO_{2.9}$.

Materials	Structure	Typical Surface	Lattice Parameters	
			Exp	*Theory*
WO_3	Monoclinic	(001)	a =7.285, b=7.517, c=3.835<br>α=90, β=90, γ=90	a=7.497, b=7.726, c=3.888<br>α=90, β=90, γ=90.18
$WO_{2.9}$	Monoclinic	(010)	a=12.05, b=3.767, c=23.590<br>α=90, β=94.72, γ=90	a=12.20, b=3.799, c=23.839<br>α=90, β=94.81, γ=90

Supplementary Note 1: Turnover frequency (TOF) calculation of the catalyst.

Molar mass – 230.24 g/mol

Density – 7.165 g/cm^3

Molar Volume – 32.13 mL/mol

Volume of a 100 nm sphere – 5.23 × 10^{-16} cm^3

Surface area of a 100 nm sphere – 3.14 × 10^{-10} cm^2

Loading amount of catalyst – 0.285 mg/cm^2

Current density at -100 mV overpotential – 0.025 A/cm^2

Current density at -200 mV overpotential – 0.077 A/cm^2

Surface area per milligram of 100 nm sphere (BET value of 48.3 cm^2/mg):

$$\frac{3.14 \times 10^{-10}\ cm^2}{1\ particle} \times \frac{1\ particle}{5.23 \times 10^{-16}\ cm^3} \times \frac{1\ cm^3}{7.165\ g} = 83.7\ cm^2/mg \qquad (1)$$

Average surface atoms per square centimeter (used for BET-based calculations also):

$$\left(\frac{1 \times 6.022 \times 10^{23}\ atoms}{1\ mol} \times \frac{1\ mol}{32.13\ cm^3}\right)^{2/3} = 7.06 \times 10^{14}\ atoms/cm^2 \qquad (2)$$

Surface Atoms per testing area (BET value = 9.69 × 10^{15} atoms/cm^2):

$$\frac{0.285\ mg}{1\ cm^2} \times \frac{83.7\ cm^2}{1\ mg} \times \frac{7.06 \times 10^{14}\ atoms}{1\ cm^2} = 1.68 \times 10^{16}\ atoms/cm^2 \qquad (3)$$

Turnover frequency (per surface atom) at η = 100 mV:

$$\frac{0.025\ A}{2 \times 1\ cm^2} \times \frac{6.022 \times 10^{23}}{96485\ C} \times \frac{1\ cm^2}{1.68 \times 10^{16}\ atoms} = 4.64\ s^{-1}\ atom^{-1} \qquad (4)$$

-100 mV overpotential (theoretical value) − 4.64 s^{-1} $atom^{-1}$

-100 mV overpotential (BET-based value) − 8.04 s^{-1} $atom^{-1}$

-200 mV overpotential (theoretical value) − 14.29 s^{-1} $atom^{-1}$

-200 mV overpotential (BET-based value) − 24.76 s^{-1} $atom^{-1}$

Supplementary Note 2: Computational details.

HER reaction mechanism and activity evaluation.

With respect to the HER in acid electrolyte ($2H^+ + 2e^- \rightarrow H_2$), the general consensus of the reaction mechanism can be described as follows[1, 2]: firstly, proton in the aqueous solution receives an electron and adsorbs on the catalyst surface ($H^+_{(aq)} + e^- + * \rightarrow H*$); Subsequently, two surface adsorbed H* can couple and desorb into H_2 ($2H* \rightarrow H_2 + 2*$) following the Tafel mechanism; alternatively, H* could also directly react with proton in the solution to produce H_2 ($H* + H^+_{(aq)} + e^- \rightarrow H_2 + *$) following the Heyrovsky mechanism. To evaluate the activity trend, it has been revealed that the adsorption energy of H atom (E_{ad}^H) inherently determines the free energy of these two processes and plays a crucial role in determining the whole catalytic activity. It is generally accepted that the relation between the exchange current density and ΔG_H (the free energy change of the discharge step, $\Delta G_H \approx E_{ad}^H + 0.20$) on the electrode would result in a volcano plot with the maximum near $\Delta G_1 = 0$ eV (at $U_{work} = U_{SHE}$)[3-7]. Qualitatively, if the H adsorption energy is evidently weak, the generation of surface H* would be hindered; while it is too strong, the removal of H* to form H_2 would be difficult, being limited by the large energy requirement. In other words, the adsorption energy of H (E_{ad}^H) can serve as a simple, yet powerful descriptor to estimate the catalytic activity.

Description of the models for $WO_3(001)$ and $WO_{2.9}(010)$.

As indicated in the XRD and STEM characterizations, both the synthesized $WO_{2.9}$ and the commercial WO_3 are crystalized in monoclinic phase, respectively. Upon full optimization of the bulk structure, their respective most stable surface, i.e. $WO_{2.9}(010)$ and $WO_3(001)$, was cleaved to

serve as the model. All the calculations were performed with Perdew-Burke-Ernzerhof (PBE) functional within the generalized gradient approximation.

Firstly, the monoclinic WO_3 is investigated as the reference. WO_3 has a series of polymorphs, and all of them possess a similar structure characteristic with the difference lying in different WO_6 octahedral tilting and the W atomic displacement. The monoclinic WO_3 shows a simple and regular pattern (Supplementary Fig. 10). Specifically, it contains six- (two-) coordinated W (O) atoms, and the W atoms is located at the center of a series of WO_6 octahedron (sharing the corner atom) while the bridging O atoms are in an approximately linear configuration ($\angle$W-O-W=173°). Along the (001) direction, the W-O bonds give a long-short alternation, exhibiting a layer structure (Supplementary Fig. 10).

With the (001) surface, monolayer O- or WO_2-termination can form in principle, forming a $(O\text{-}WO_2)_n$ slab (Supplementary Fig. 11). However, the monolayer O termination would lead to an energetically unstable polar surface causing reconstruction. The reconstruction of the (001) surface of the idealized simple WO_3 has been well studied, and the $(\sqrt{2}\times\sqrt{2})$R45° reconstruction observed experimentally was indicated to be the most stable pattern theoretically, in which half of the surface oxygen atoms alternatively along the [100] and [010] direction are transferred from the top to the bottom layer, forming a nonpolar $[O\text{-}(WO_2)_2\text{-}O]_n$ slab. Herein we would focus on this surface termination for the monoclinic WO_3(001) surface (see Fig. 4 and Supplementary Fig. 11).

Detailed binding energy study on WO_3(001) and $WO_{2.9}$(010).

As shown in Fig. 4a, the WO_3(001) surface exposes five-coordinated W atom, one-coordinated terminal O atom and the two-coordinated bridge O, denoted as W_{5c}, O_t and O_{bri}, respectively. On the surface W_{5c} of the pure WO_3(001), the bonding with H is very weak with a W-H bond length at ~1.73 Å, giving an adsorption energy as low as 1.20 eV, which evidently deviates from the optimal

adsorption strength, indicating the low catalytic activity. By contrast, atomic H can readily adsorb at the terminal O_t with an adsorption energy as high as -1.42 eV. However, it can be expected that such kind of H would hardly desorb to form H_2 and results in low activity. Similarly, with respect to the lattice bridge oxygen, the H adsorption is also too strong with E_{ad}^H being as large as -0.70 eV (ΔG_H = -0.50 eV), indicating to be inert either.

On the other hand, as a reduced phase of WO_3, monoclinic $WO_{2.9}$ exhibits a new kind of topological configuration locally relative to WO_3, which could be considered as a result of reconstruction of monoclinic WO_3 in the reduction process by translating the nearest two-row WO_6 octahedrons every three ones along the [100] or [010] direction, forming a collection of edge-shared WO_6 octahedron from the original corner-shared interconnection type. Similar with WO_3(001), $WO_{2.9}$(010) can be terminated by O or WO layer, and to cancel the diploe effect, half of the surface terminal O along the [90-4] and [409] direction approximately every other one are transferred to the bottom layer (Fig. 4b and Supplementary Fig. 11). Herein, we examined two kinds of possible reconstruction configurations (Supplementary Fig. 12). To systematically examine the binding ability of $WO_{2.9}$(010) surface with the stable config_1, various possible W_{5c} sites were checked, including the sites S_N (N = 1, 2, 3) in the characteristic region and a series of reference sites distributing outside this region (denoted as R_N (N = 1, 2, …, 6)), as shown in Fig. 4b and Supplementary Fig. 13. As shown in Supplementary Table 3, it is interesting that the adsorption energy on all these sites is largely enhanced relative to WO_3(001), and site S_N (N = 1, 2, 3) in the characteristic region as well as the reference site (R1, R2, R3) nearest to the region exhibit the strongest binding ability with the order of -0.1 eV, while the farest one (R6) from the region gives the weakest binding ability (E_{ad}^H = 0.46 eV). For example, the adsorption energy at the S3 site is calculated to be -0.19 eV, and accordingly, the free energy change of the discharge step (H^+

+ e$^-$ → H*) for HER at the standard condition (U = 0 V *vs* U_{SHE}, pH = 0) is calculated to be 0.01 eV, fulfilling the ΔG_H = 0 eV requirement, and thus its high catalytic activity can be expected. Besides the W_{5c} site, the catalytic activity of the terminal O on $WO_{2.9}(010)$ were also examined. Four kinds of one-coordinated terminated oxygen, denoted as O_I, O_{II}, O_{III} and O_{IV}, respectively, are selected as demonstration (Supplementary Fig. 12). The adsorption energies were calculated to be -0.98 eV, -0.98 eV, -0.74 eV and -0.88 eV, respectively, corresponding to ΔG_H = -0.50 ~ -0.74 eV, indicating their low catalytic activity (Fig. 4c) due to the too strong binding ability. Similar with $WO_3(001)$, these formed terminal OH could further adsorb H and form H_2O, resulting in the possible reduction. We thus also consider the surface reduction by removing all the terminal O from the $p(1\times1)$ $WO_{2.9}(010)$ slab, corresponding to a W/O ratio of $W_{60}O_{154}$. Twelve representative adsorption sites (denoted as S_i (i = 1, 2, 3) and R_i (i = 1, 2, …, 9)) were considered (Supplementary Fig. 13). It is found that the adsorption energy were further improved by the order of only ~0.30 eV compared with clean $WO_{2.9}(010)$ surface (Supplementary Table 4). From Fig. 4c, one can see that the activity can remain at the high level, despite being a little lower to some extent relative to clean $WO_{2.9}(010)$. Therefore, it can be rationalized that $WO_{2.9}$ exhibits a high and stable activity. As illustrated above, the improved H adsorption ability at the surface W_{5c} largely contribute the high catalytic activity of $WO_{2.9}(010)$ relative to $WO_3(001)$.

Detailed electronic analysis of $WO_3(001)$ and $WO_{2.9}(010)$.

The W_{5c}-H bond exhibits evident covalent bond on both surfaces (Supplementary Fig. 14), mainly ascribed to the overlapping between the W_{5c} $d_Z{}^2$ orbital and H_{1s} orbital. Projected density of state on the *d*-orbital (*d*-PDOS) of the surface W_{5c} on $WO_{2.9}(010)$ and $WO_3(001)$ were analyzed, in which a series of W_{5c} cations on $WO_{2.9}(010)$ were considered and the site S_1 for $WO_{2.9}(010)$ was taken as a demonstration. As shown in Supplementary Fig. 14, $WO_3(001)$ has an evident band

gap and the VBM is below the Fermi level by ~1 eV, which therefore disfavor the electron transfer from W_{5c} to H and go against the further orbital overlapping. In contrast, with respect to $WO_{2.9}$(010), the PDOS of the surface W_{5c} shows that there appear a new *d* band across the Fermi level, indicating an evident metallic properties, which largely increase the *d*-band energy level near the Fermi level, and facilitate the bonding of W_{5c} and H. The PDOS of other W_{5c} atom on the $WO_{2.9}$(010) surface were also given (Supplementary Fig. 15), from which we can see that near the Fermi level, all of them appear an occupied *d*-band with the center being in the range E_F-1 ~ E_F. According to the frontier orbital theory, approximately we calculated the *d*-band center (ε_d) of various W_{5c} site of $WO_{2.9}$(010) within this band region ranging from E_F-2 to E_F, as well as the monoclinic WO_3(001) surface, and examined the dependence of the adsorption energy on the ε_d, which indeed shows a close linear correlation (R^2 = 0.97, Supplementary Fig. 16). Therefore, it further indicates the highest occupied *d*-orbital of surface W_{5c} largely affects the binding ability toward H atom, and the appearance of *d*-band around the Fermi level for $WO_{2.9}$(010) is an important factor for the strengthened binding ability compared to WO_3. In addition, the calculated work function of $WO_{2.9}$(010) and WO_3(001) suggests that $WO_{2.9}$ has a high Fermi level by 0.70 eV, and may thus facilitate the reduction process to occur kinetically.

Supplementary References

1. Tilak, B. V., Ramamurthy, A. C. & Conway, B. E. High performance electrode materials for the hydrogen evolution reaction from alkaline media. *Proc. Indian Acad. Sci.* **97**, 359-393 (1986).

2. Appleby, A. J., Kita, H., Chemla, M. & Bronoel, G. in Encyclopedia of Electrochemistry of the Elements, A. J. Bard, Eds. (Marcel Dekker, New York, 1982), vol. 9, part A, pp. 383-597.

3. Bockris, J. O'M., Reddy, A. K. N. & Gamboa-Aldeco, M. Modern Electrochemistry (Kluver Academic/Plenum Publishers, New York, 2000), pp. 1285-1286. [second edition]

4. Nørskov, J. K. *et al.* Trends in the exchange current for hydrogen evolution. *J. Electrochem. Soc.* **152**, J23-J26 (2005).

5. Schmickler, W. & Trasatti, S. Comment on "Trends in the exchange current for hydrogen evolution". *J. Electrochem. Soc.* **153**, L31-L32 (2006).

6. Xing, J. *et al.* Active sites on hydrogen evolution photocatalyst. *J. Mater. Chem. A* **1**, 15258-15264 (2013).

7. Xing, J. *et al.* Stable isolated metal atoms as active sites for photocatalytic hydrogen evolution. *Chem. Eur. J.* **20**, 2138-2144 (2014).
